# Caspase-4/11 exacerbates disease severity in SARS–CoV-2 infection by promoting inflammation and immunothrombosis

**DOI:** 10.1073/pnas.2202012119

**Published:** 2022-05-19

**Authors:** Mostafa M. Eltobgy, Ashley Zani, Adam D. Kenney, Shady Estfanous, Eunsoo Kim, Asmaa Badr, Cierra Carafice, Kylene Daily, Owen Whitham, Maciej Pietrzak, Amy Webb, Jeffrey Kawahara, Adrian C. Eddy, Parker Denz, Mijia Lu, KC Mahesh, Mark E. Peeples, Jianrong Li, Jian Zhu, Jianwen Que, Richard Robinson, Oscar Rosas Mejia, Rachael E. Rayner, Luanne Hall-Stoodley, Stephanie Seveau, Mikhail A. Gavrilin, Xiaoli Zhang, Jeronay Thomas, Jacob E. Kohlmeier, Mehul S. Suthar, Eugene Oltz, Andrea Tedeschi, Frank H. Robledo-Avila, Santiago Partida-Sanchez, Emily A. Hemann, Eman Abdelrazik, Adriana Forero, Shahid M. Nimjee, Prosper N. Boyaka, Estelle Cormet-Boyaka, Jacob S. Yount, Amal O. Amer

**Affiliations:** ^a^Department of Microbial Infection and Immunity, The Ohio State University College of Medicine, Columbus, OH 43210;; ^b^Neuroscience graduate program, The Ohio State University, Columbus, OH 43210;; ^c^Infectious Diseases Institute, The Ohio State University, Columbus, OH 43210;; ^d^Faculty of Pharmacy, Helwan University, Cairo,11731 Egypt;; ^e^Department of Veterinary Biosciences, The Ohio State University, Columbus, OH 43210;; ^f^Department of Biomedical Informatics, The Ohio State University, Columbus, OH 43210;; ^g^Center for Vaccines and Immunity, Abigail Wexner Research Institute at Nationwide Children’s Hospital, Columbus, OH 43205;; ^h^Department of Pathology, The Ohio State University, Columbus, OH 43210;; ^i^Division of Digestive and Liver Diseases and Center for Human Development, Department of Medicine, Columbia University, New York, NY 10027;; ^j^Department of Microbiology and Immunology, Emory University, Atlanta, GA 30322;; ^k^Department of Pediatrics, Emory University School of Medicine, Atlanta, GA 30322;; ^l^Emory Vaccine Center, Yerkes National Primate Research Center, Emory University, Atlanta, GA 30322;; ^m^Department of Neuroscience, Chronic Brain Injury Discovery Theme, The Ohio State University, Columbus, OH 43210;; ^n^Center for Microbial Pathogenesis, Abigail Wexner Research Institute at Nationwide Children’s Hospital, Columbus, OH 43205;; ^o^Center for Informatics Science, Nile University, Giza, 12525, Egypt;; ^p^Department of Neurological Surgery, The Ohio State University, Columbus, OH 43210

**Keywords:** innate immunity, SARS–CoV-2, thrombosis

## Abstract

Severe acute respiratory syndrome coronavirus 2 (SARS–CoV-2) is a worldwide health concern, and new treatment strategies are needed. Targeting inflammatory innate immunity pathways holds therapeutic promise, but effective molecular targets remain elusive. Here, we show that human caspase-4 (CASP4) and its mouse homolog, caspase-11 (CASP11), are up-regulated in SARS–CoV-2 infections and that *CASP4* expression correlates with severity of SARS–CoV-2 infection in humans. SARS–CoV-2–infected *Casp11*^−/−^ mice were protected from severe weight loss and lung pathology, including blood vessel damage, compared to wild-type (WT) mice and mice lacking the caspase downstream effector gasdermin-D (*Gsdmd^−/−^*). Notably, viral titers were similar regardless of CASP11 knockout. Global transcriptomics of SARS–CoV-2–infected WT, *Casp11*^−/−^, and *Gsdmd^−/−^* lungs identified restrained expression of inflammatory molecules and altered neutrophil gene signatures in *Casp11*^−/−^ mice. We confirmed that protein levels of inflammatory mediators interleukin (IL)-1β, IL-6, and CXCL1, as well as neutrophil functions, were reduced in *Casp11*^−/−^ lungs. Additionally, *Casp11*^−/−^ lungs accumulated less von Willebrand factor, a marker for endothelial damage, but expressed more Kruppel-Like Factor 2, a transcription factor that maintains vascular integrity. Overall, our results demonstrate that CASP4/11 promotes detrimental SARS–CoV-2–induced inflammation and coagulopathy, largely independently of GSDMD, identifying CASP4/11 as a promising drug target for treatment and prevention of severe COVID-19.

## Significance

We report the discovery of fundamental roles for the noncanonical inflammasome molecule Caspase-4/11 in promoting pathological inflammatory and prothrombotic pathways in severe acute respiratory syndrome coronavirus 2 (SARS–CoV-2) infections. Our work demonstrates that Caspase-11 has a broader role in immune responses beyond its previously appreciated effects in bacterial infections. Further, we show that Caspase-11–deficient mice infected with SARS–CoV-2 fare significantly better in terms of overall illness, lung inflammation, and thrombosis than wild-type (WT) mice, thus implicating Caspase-11 as a new therapeutic target for preventing or treating COVID-19.

Severe acute respiratory syndrome coronavirus 2 (SARS–CoV-2) is the causative infectious agent of the worldwide COVID-19 pandemic (1). SARS–CoV-2 is a positive sense single-stranded RNA virus that can induce hyperinflammatory responses, including cytokine storm, in the lungs as well as extrapulmonary organs in severe cases (2). Interleukin (IL)-6, CXCL1, IL-1α, IL-1β, and type I interferons (IFNs), among other cytokines, contribute to pathological manifestations of the SARS–CoV-2 infection (3). In addition, formation of thrombi that can cause myocardial infarction, stroke, and pulmonary embolism is a hallmark of severe COVID-19. Endothelial and neutrophil dysfunctions during SARS–CoV-2 infection increase the incidence of thromboembolic complications (4), which are initiated by von Willebrand factor (VWF), a glycoprotein released by damaged endothelial cells and megakaryocytes (5, 6). VWF also self-associates, forming strings protruding into the lumen that serve as a scaffold for platelet adhesion and aggregation (6). Cellular sensors of infection, such as Toll-like receptor 2, C-type lectin receptors, and the NLRP3 inflammasome have been implicated in triggering the induction and secretion of cytokines and inflammatory lung damage in SARS–CoV-2 infections (7). However, the contribution of these pathogen-sensing pathways and other inflammasome components in mediating host defense versus immune-mediated pathology and thrombosis during SARS–CoV-2 infection in vivo remains unclear (7). While effector molecules downstream of infection-sensing pathways, such as specific inflammatory cytokines, have been targeted in attempts to limit virus-induced tissue damage, most of these strategies failed to exert major benefits in human clinical trials (8). Therefore, strategies targeting molecules upstream of multiple inflammatory cytokines or chemokines may be more effective, though this remains to be experimentally tested. Here, we investigated the role of a major member of the noncanonical inflammasome, caspase-11 (CASP11), and its downstream effector Gasdermin-D (GSDMD) in SARS–CoV-2 infection and disease severity using knockout mouse models and mouse-adapted SARS–CoV-2.

Caspases are a family of cysteine proteases that specifically cleave their substrates at the C-terminal side of aspartic acid residues. CASP11 is a murine protein that is critical for defense against bacterial pathogens. Human caspase-4 (CASP4) and caspase-5 (CASP5) display high homology to murine CASP11 (9, 10), and we have demonstrated that human CASP4 mediates many functions of mouse CASP11 in macrophages during bacterial infections (9). CASP4/11 is a component of the noncanonical inflammasome with multiple functions that remain to be fully characterized. One major role for this protein is the cleavage of GSDMD (11). Once cleaved, the GSDMD N-terminal fragment inserts into the plasma membrane of eukaryotic cells to form pores that allow the release of IL-1β and other molecules and that can lead to cell lysis and death, known as pyroptosis (12). Interestingly, roles for GSDMD downstream of caspases have been posited to mediate inflammatory pathology during SARS–CoV-2 infection (13), though clinical trials testing inhibitors of GSDMD in COVID-19 patients were not promising (8) and SARS–CoV-2 infection studies in GSDMD genetically deficient animal models have not yet been performed. Likewise, the role of CASP4/11 in viral infections has not been explored, despite the induction of these proteins by the antiviral type I and II IFNs (8). This notable induction by IFNs and the broad roles of CASP4/11 in regulating diverse inflammatory pathways (10) prompted us to investigate its role in SARS–CoV-2 infections.

## Results

### CASP4/11 Expression is Elevated in Lungs during SARS–CoV-2 Infections of Mice and Humans and Correlates with Disease Severity in Humans.

**Fig. 1.**
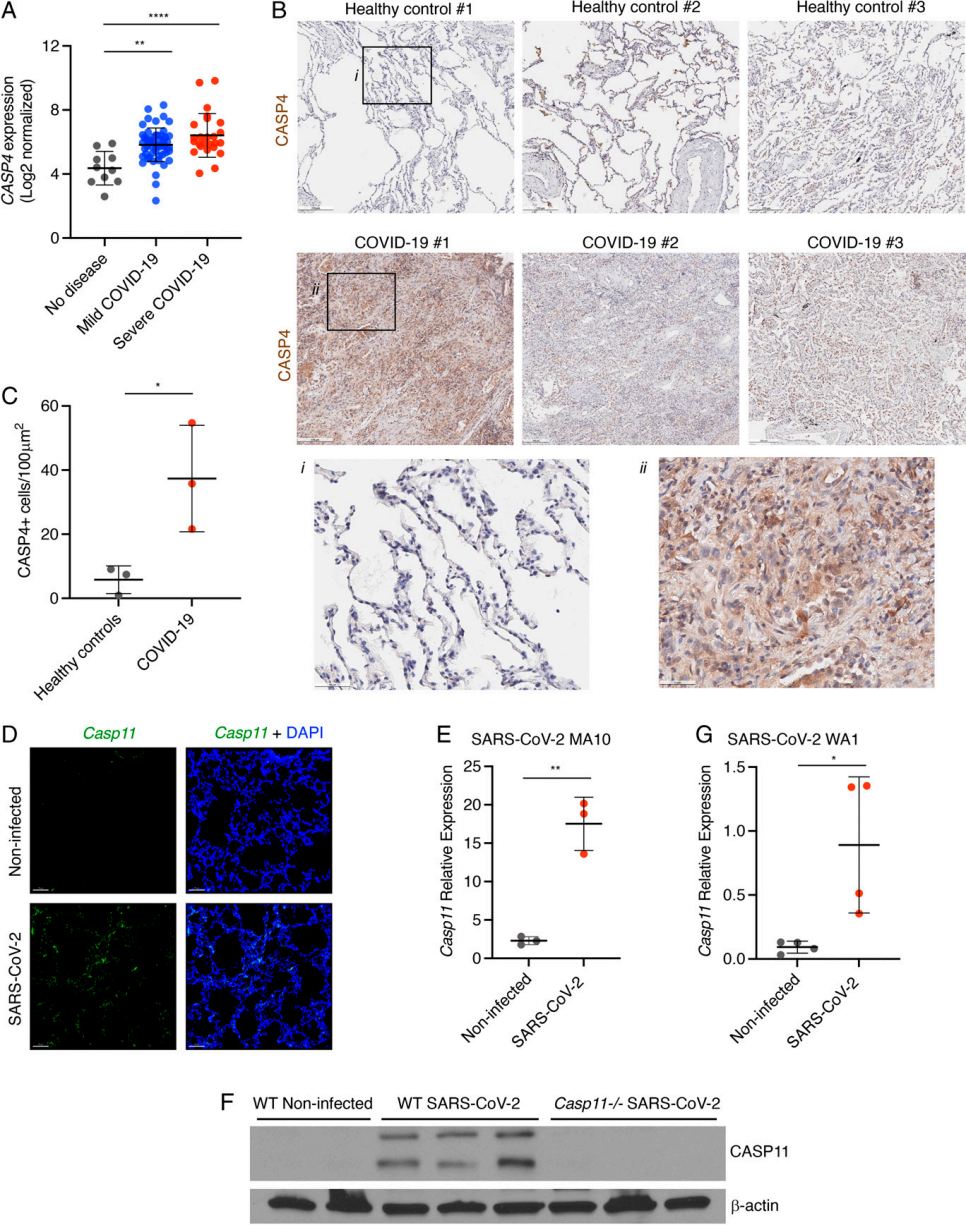
CASP4 is up-regulated in humans and mice infected with SARS–CoV-2. (*A*) *CASP4* expression levels from RNA sequencing of nasopharyngeal swab samples from patients with no disease, mild SARS–CoV-2, or severe SARS–CoV-2 [GSE145926]; one-way ANOVA with Tukey’s multiple comparisons test. (*B*) Human lung samples from three donors with healthy lungs or from three donors who died of SARS–CoV-2 were stained for CASP4 (brown). (*B*, *i* and *ii*) Black boxes outline zoomed regions. Scale bars represent 200 μm (*B*) and 50 μm (i and ii). (*C*) Quantification of CASP4-positive cells from lungs in *B*; unpaired *t* test. (*D*–*F*) Mice were infected for 4 d with mouse-adapted SARS–CoV-2 (MA10, 10^5^ pfu). (*D*) *Casp11* RNA (green, RNAscope ISH) and DAPI (blue) were visualized (3D intensity projection image) in lung sections using 20x objective, scale bars represent 10 μm. (*E*) *Casp11* RNA levels were quantified in lung samples (*n* = 3) by qRT-PCR; unpaired *t* test. (*F*) CASP11 protein levels in lungs described in *D* (*n* = 3) were examined by Western blot. (*G*) K18-hACE2 mice were infected for 4 d with human SARS–CoV-2 (WA1, 10^5^ pfu), and *Casp11* RNA levels were quantitated in lung samples (*n* = 4) by qRT-PCR; unpaired *t* test. Error bars in *A*, *C*, *E*, and *G* represent SD of the mean. *P < 0.05, **P < 0.005, ****P < 0.0001.

**Fig. 2.**
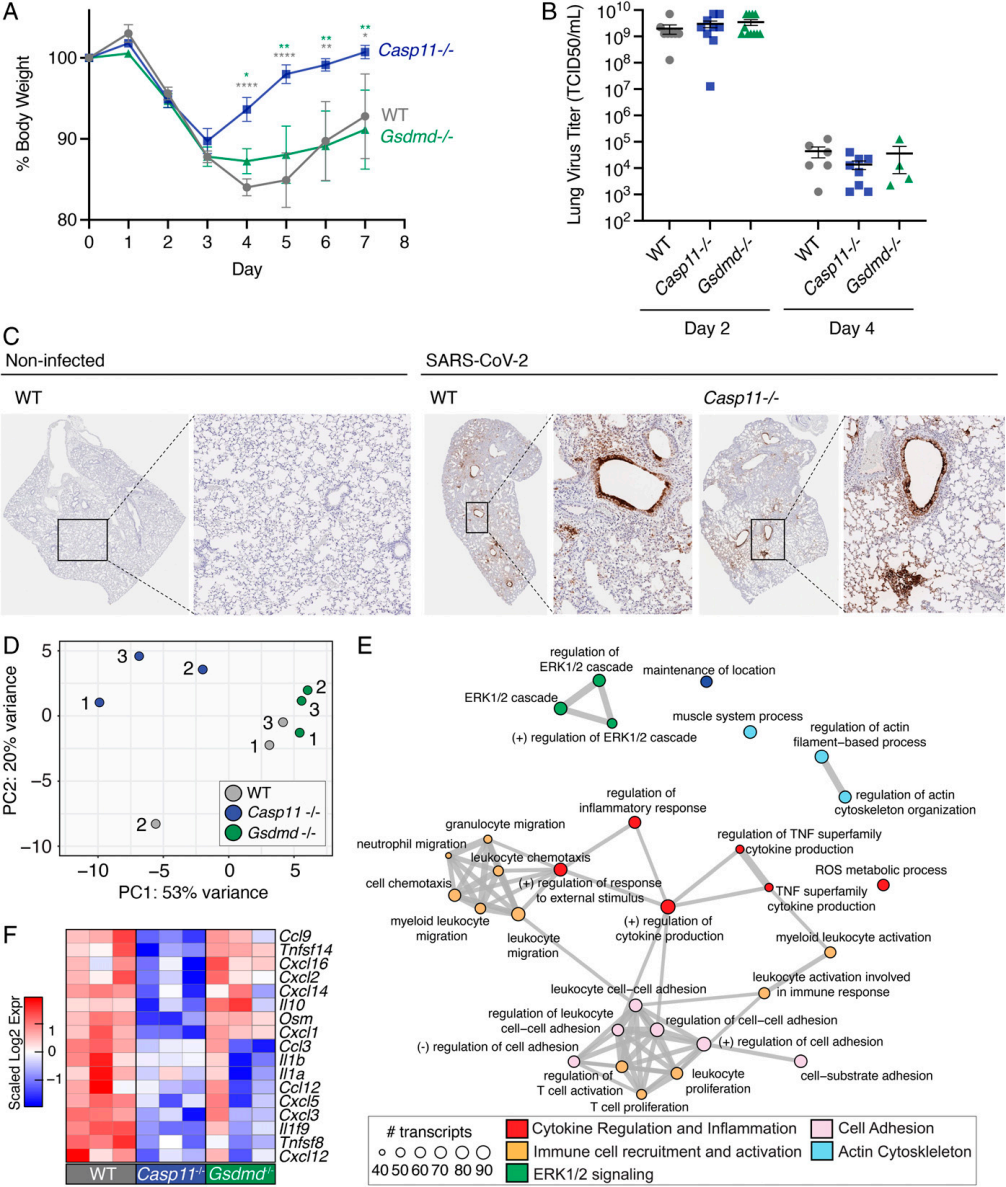
*Casp11*^−/−^ mice show decreased SARS–CoV-2 infection severity without affecting viral titers but by modulating specific inflammatory programs. (*A*–*C*) WT, *Casp11^−/−^*, and *Gsdmd^−/−^* mice were infected with SARS–CoV-2 (MA10, 10^5^ pfu). (*A*) Weight loss was tracked for 7 d. *P < 0.05, **P < 0.005, ****P < 0.0001; ANOVA with Bonferroni’s multiple comparisons test, day 0 to 4 WT (*n* = 7), *Casp11^−/−^* (*n* = 10), *Gsdmd^−/−^* (*n* = 9); day 5 to 7 WT (*n* = 4), *Casp11^−/−^* (*n* = 7), *Gsdmd^−/−^* (*n* = 6). Error bars in represent SEM. (*B*) TCID50 viral titers were quantified in lung tissue homogenates. Error bars represent SD of the mean. (*C*) Sections from noninfected control lungs or lungs collected at 4 d after infection were stained for SARS–CoV-2 nucleocapsid protein (brown staining, images representative of at least three mice per group). Insets outline zoomed regions. (*D–F*) WT, *Casp11^−/−^*, and *Gsdmd^−/−^* mice (*n* = 3) were infected with SARS–CoV-2 (MA10, 10^5^ pfu) for 2 d. RNA was extracted from lungs and subjected to RNA sequencing. (*D*) PCA of SARS–CoV-2–infected lung gene expression with points representing individual WT (gray), *Casp11*^−/−^(blue), and *Gsdmd*^−/−^ (green) mice. PC1 and PC2 represent principal component1 and 2, respectively. (*E*) Top 30 significant Gene Ontology Biological Pathways are depicted. Node size indicates the number of transcripts within each functional category. Edges connect overlapping gene sets. Numbers represent individual replicates, and color indicates relative up-regulation (red) or down-regulation (blue) in gene expression. TNF: tumor necrosis factor alpha, ROS: reactive oxygen species. (*F*) Heatmap of significantly changed cytokine and chemokine genes when comparing *Casp11*^−/−^ infected lungs versus WT. Expression scaling is relative to WT and *Gsdmd^−/−^* mice for comparisons (*n* = 3) (*P* < 0.05).

**Fig. 3.**
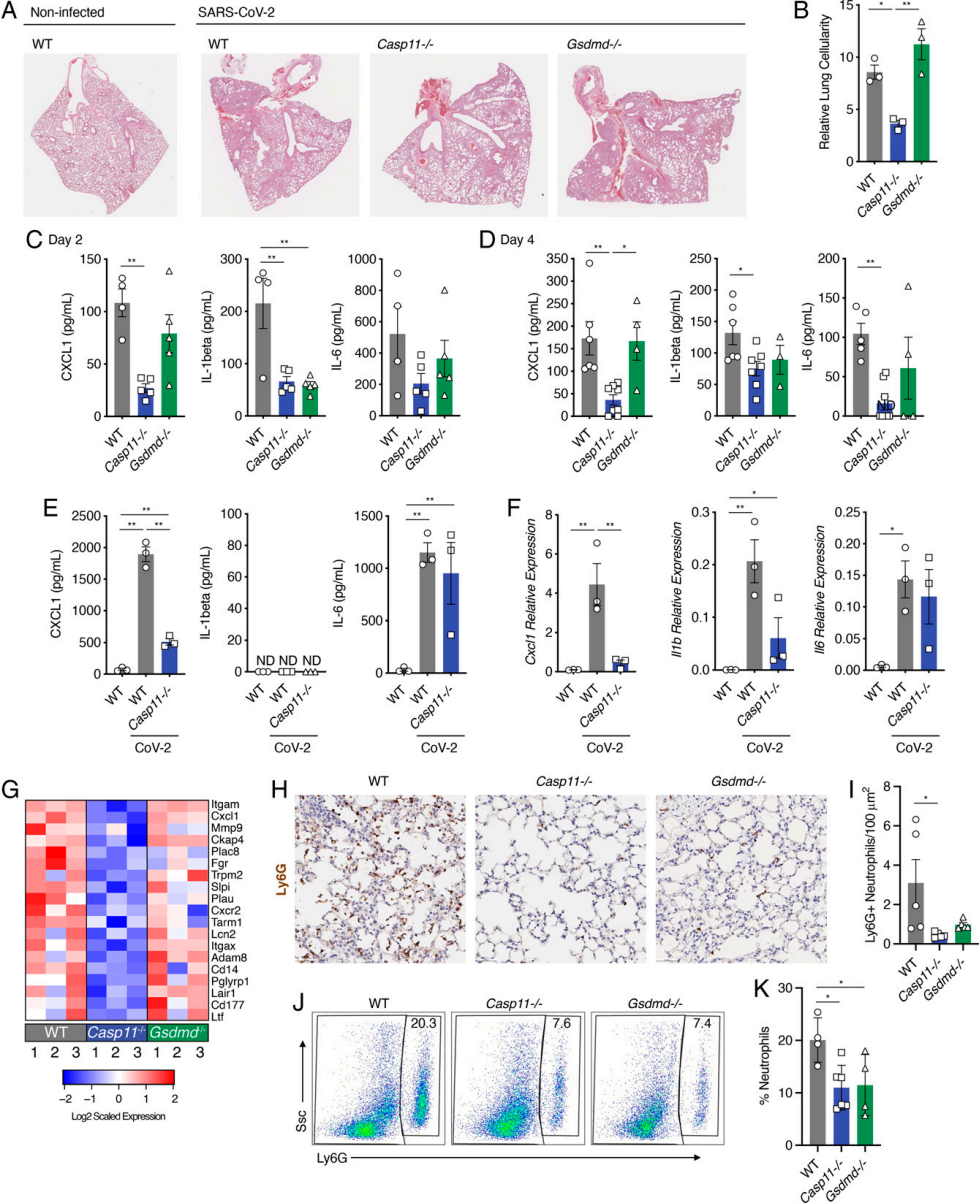
*Casp11^−/−^* mice show decreased lung inflammation, less neutrophil recruitment, and altered neutrophil function in response to SARS–CoV-2 infection. (*A* and *B*) WT and *Casp11^−/−^* mice were infected with SARS–CoV-2 (MA10, 10^5^ pfu). (*A*) Lung sections from day 4 after infection were stained with H&E to visualize lung damage and airway consolidation. (*B*) Lung sections as in *A* were analyzed by the color deconvolution method to quantify cellularity as an indicator of cellular infiltration and alveolar wall thickening; ANOVA with Tukey’s multiple comparisons test. (*C* and *D*) Lung homogenates from 2 or 4 d after infection were analyzed by ELISA for detection of CXCL1, IL-1β, or IL-6; ANOVA with Tukey’s multiple comparisons test. (*E* and *F*) Macrophages were purified from lungs of mice of the indicated genotype. The cells were infected with mouse-adapted SARS–CoV-2 (multiplicity of infection [MOI] 1 for 24 h). Cell supernatants were analyzed by ELISA, or cellular RNA was analyzed by qRT-PCR for the indicated chemokine/cytokines; ANOVA with Tukey’s multiple comparisons test. (*G*) Heatmap of significantly changed neutrophil-related genes comparing *Casp11*^−/−^ infected lungs versus WT (*P* < 0.05). Expression scaling is relative to WT and *Gsdmd^−/−^* mice for comparisons. Numbers represent individual replicates, and color indicates relative up-regulation (red) or down-regulation (blue) in gene expression. (*H* and *I*) Lung sections of day 2 SARS–CoV-2–infected WT, *Casp11*^−/−^, and *Gsdmd^−/−^*mice (*n* = 5) stained with neutrophil marker Ly6G (*H*) and quantified in *I*. (*J* and *K*) Flow cytometry of lung single-cell suspensions previously gated on CD45^+^ cells from WT (*n* = 4), Casp11^−/−^ (*n* = 6), and Gsdmd^−/−^ (*n* = 4) mice (*J*) as in *H* and quantified in *K*. All error bars represent SEM. *P < 0.05, **P < 0.005.

CASP4/11 is reported to be widely expressed in epithelial and endothelial cells, where it is involved in the response of these cells to bacterial lypopolysacchride (LPS) (14, 15). CASP4/11 also has well-characterized roles in responding to gram-negative and -positive bacteria in immune cells, including macrophages and neutrophils (16, 17). To gain a better understanding of CASP4 expression patterns, we examined human lung single-cell RNA sequencing (scRNAseq) data for CASP4 expression. These data show CASP4 transcripts in all lung cell types including type I and type II pneumocytes, ciliated cells, and club cells and suggested particularly prominent expression in macrophages and endothelial cells (*SI Appendix*, Fig. 1*A*) (18). Expression of the related protein CASP5 was restricted to macrophages (*SI Appendix*, Fig. 1*B*) (18). While CASP4/11 can be expressed in resting cells, it is highly induced in response to bacterial infections (19). The analysis of publicly available RNA sequencing data of nasopharyngeal swab material from subjects with SARS–CoV-2 and healthy donors (Gene Expression Omnibus [GEO] accession No. GSE163151) revealed that *CASP4* is highly expressed in the airway of SARS–CoV-2–infected patients and that expression levels increase with disease severity (Fig. 1*A*). CASP5 expression was also up-regulated in infected samples, while CASP7 and CASP10 expression served as controls that were unaffected by infection status (*SI Appendix*, Fig. 2). Additionally, we found that human lung sections from COVID-19 patients show higher levels of CASP4 staining compared with healthy lung controls (Fig. 1*B*), owing to greater numbers of CASP4-positive cells in the infected lung tissue (Fig. 1*C*). We then performed intranasal infection of C57BL/6 wild-type (WT) mice with pathogenic mouse-adapted SARS–CoV-2 (strain MA10) (20) and found that infection strongly induces *Casp11* expression throughout murine lung tissue within 4 d of infection, as detected by RNAscope in situ hybridization (ISH) (Fig. 1*D*) and confirmed by qRT-PCR (Fig. 1*E*). The level of CASP11 protein likewise went from low to highly expressed in response to SARS–CoV-2 infection of murine lungs (Fig. 1*F*). We further examined infection of K18-hACE2 mice expressing the human ACE2 receptor using human isolate SARS–CoV-2 strain USA-WA1/2020 (WA1). Similar to mouse-adapted SARS–CoV-2, the nonadapted human virus strongly induced the lung expression of CASP11 as demonstrated by qRT-PCR (Fig. 1*G*). These results are additionally supported by analysis of scRNAseq of mouse lung cells, which similarly identified wide expression of CASP11 that is sustained or enhanced in most cell types following SARS–CoV-2 infection, including high CASP11 expression in inflammatory cells recruited to the lung following infection (*SI Appendix*, Fig. 3) (21). Overall, CASP4 is highly expressed in the lungs of COVID-19 patients, and CASP11 is similarly increased upon SARS–CoV-2 infection of mice.

### *Casp11* Deficiency Reduces Disease Severity in SARS–CoV-2–Infected Mice.

We next examined whether CASP11 regulates disease severity caused by SARS–CoV-2 infection. WT, *Casp11*^−/−^, and *Gsdmd*^−/−^ mice were infected with SARS–CoV-2 MA10 for comparison of weight loss, a commonly used indicator of overall infection severity in mice (22). We found that WT mice lost a significant percentage of their body weight between days 1 and 4 after infection, followed by partial recovery of weight up to day 7, at which point we ended our experiments (Fig. 2*A*). *Casp11*^−/−^ mice, on the other hand, lost weight only up to day 3 and then rapidly recovered fully to their original weight by day 5 (Fig. 2*A*). In comparison, weight loss of *Gsdmd*^−/−^ mice was not significantly different from that of WT mice (Fig. 2*A*). These data indicate that CASP11 promotes disease severity during SARS–CoV-2 infection and that this function is not mediated by GSDMD.

To determine whether differences in disease severity could be explained by differences in viral replication, we quantified live virus titers in WT, *Casp11*^−/−^, and *Gsdmd*^−/−^ mouse lungs at 2 and 4 d after infection. We found that viral loads were similar with no statistical difference between the groups at either time point (Fig. 2*B*). We also observed, in agreement with previous reports (23), that viral titers were decreased at day 4 compared with day 2 in all groups, demonstrating that neither CASP11 nor GSDMD is required for viral clearance mechanisms in mice (Fig. 2*B*). To corroborate these findings, lung sections from WT and *Casp11*^−/−^ mice were stained for SARS–CoV-2 nucleocapsid protein, and similar staining patterns were observed with prominent infection of cells lining the airways and neighboring alveoli (Fig. 2*C*). Overall, these results demonstrate that loss of CASP11, but not GSDMD, prevents severe disease in SARS–CoV-2 infection without affecting virus replication or clearance.

### *Casp11^−/−^* Controls Specific Inflammatory Gene Signatures in SARS–CoV-2–Infected Lungs.

**Fig. 4.**
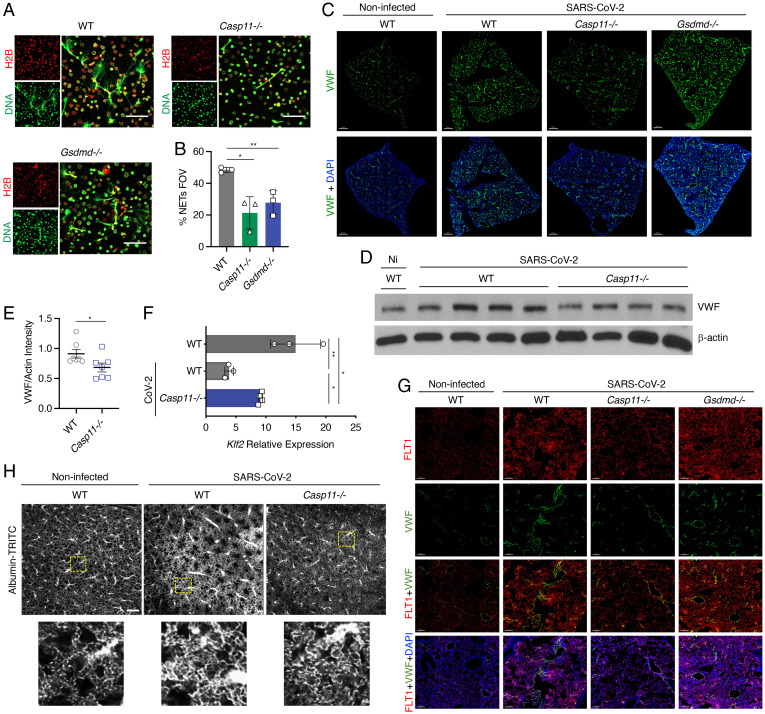
*Casp11^−/−^* neutrophils undergo less NETosis, and *Casp11^−/−^* mice show decreased indicators of coagulopathy in lungs after SARS–CoV-2 infection. (*A*) Neutrophils from WT, *Casp11*^−/−^, and *Gsdmd^−/−^* mice were treated with PMA, and NET formation was visualized by staining with anti-mouse Histone 2b (H2B) (red) and anti-dsDNA (green). Images were captured at 60x magnification. (*B*) Percentage of cells undergoing NETosis as averaged from 10 fields of view (FOVs) for each experimental replicate. Error bars represent SEM. (*C*–*G*) WT, *Gsdmd^−/−^*, and *Casp11^−/−^* mice were infected with SARS–CoV-2 (MA10, 10^5^ pfu). Lungs were collected at day 4 after infection. (*C*) RNA for *VWF* (green) was stained by RNAscope ISH, and nuclei were stained with DAPI (blue). Images were captured by a 20x objective in a 3D stitched panoramic view. Intensity projection images were created using IMARIS software. Scale bars represent 500 μm. (*D* and *E*) Western blotting of lung homogenates from noninfected WT and SARS–CoV-2–infected WT and *Casp11^−/−^* mice (*D*) as described in *C* were quantified in *E*; unpaired *t* test. Error bars represent SEM. (*F*) qRT-PCR quantification of KLF2 in the lungs of mice as described in *C*; unpaired *t* test. Error bars represent SEM. (*G*) Confocal microscopy for the colocalization of *VWF* RNA (green) with endothelial VEGF receptor subtype 1 (FLT1, red) in the lungs of mice as described in *C*. Nuclei were stained with DAPI (blue). Images were captured with a 20x objective in a z-stack 3D view and visualized using intensity projection function of IMARIS software. (*H*) Vasculature imaging of intact lungs 4 d after infection. *Below*: Higher-magnification view of the regions in yellow boxes. (Scale bar, 200 μm.) *P < 0.05, **P < 0.005.

To examine global transcriptional effects of CASP11 and GSDMD in the lung during SARS–CoV-2 infections, we infected WT, *Casp11*^−/−^, and *Gsdmd*^−/−^ mice and performed RNA sequencing on lung RNA at 2 d after infection. Day 2 was chosen because it is the peak of virus replication in the lungs of mice (20). Distinct gene signatures in WT, *Casp11*^−/−^, and *Gsdmd*^−/−^ infected lungs were seen using dimensionality reduction approaches, with the *Casp11*^−/−^ infected lung profiles showing the most extensive divergence in gene expression patterns (Fig. 2*D*). We contrasted the significant gene expression changes (*P* value <0.05) in infected *Casp11*^−/−^ and *Gsdmd*^−/−^ lungs relative to WT mice to understand how these genes impact the transcriptional landscape in terms of differentially expressed genes (either significantly up-regulated or down-regulated) (*SI Appendix*, Fig. 4*A*). Functional analysis of differentially expressed genes in *Casp11*^−/−^ versus WT lungs revealed an enrichment for genes corresponding to immunological pathways involved in cytokine production and inflammation (red), immune cell migration and activation (orange), cell adhesion (pink), and extracellular signal–related kinase (ERK)1/2 signaling (green) (Fig. 2*E*). In accordance with known actin polymerization regulation imparted by CASP11, the absence of *Casp11* in SARS–CoV-2 infection also resulted in expression changes in genes involved in actin regulatory pathways (blue) (Fig. 2*E*) (9, 16, 24). Since sensing of virus replication by cells generally induces IFN-mediated antiviral responses, we investigated whether CASP11 or GSDMD shape the antiviral gene program during SARS–CoV-2 infection. First, we specifically examined the expression of IFN-stimulated genes (ISGs), which are abundantly up-regulated by type I IFN stimulation in murine airway epithelial cells (25). Deficiency of CASP11 or GSDMD did not result in differential ISG expression relative to WT infected lungs (log2 fold change [LFC] |0.58|; *P* value <0.05) expression relative to WT infected lungs (*SI Appendix*, Fig. 4 *B* and *C*). This lack of interplay between CASP11 and GSDMD with the type I IFN antiviral pathway is consistent with the similar viral titers observed in our distinct animal groups (Fig. 2*B*).

Specific examination of cytokine and chemokine genes revealed a statistically significant down-regulation of several important inflammatory mediators in the absence of *Casp11*, including cytokines *Il1b*, *Il1α*, and *Il1f9* and chemokines *Cxcl1*, *Cxcl2*, *Cxcl14*, *Cxcl3*, *Cxcl5*, and *Ccl3* (Fig. 2*F*). These findings are consistent with ERK activation downstream of CXCL1 and CXCL3 signaling as highlighted in Fig. 2*E*. Knockout of *Gsdmd* had less impact on the magnitude of cytokine and chemokine expression compared to *Casp11* knockout (Fig. 2*F*). Overall, our results demonstrate that CASP11 controls a specific subset of inflammatory responses during SARS–CoV-2 infection.

### CASP11 Promotes the Production of Specific Inflammatory Mediators in Response to SARS–CoV-2 In Vivo and In Vitro.

To examine the role of CASP11 in mediating the pathological hallmarks of SARS–CoV-2 pulmonary infection, lung sections from infected WT, *Casp11*^−/−^, *and Gsdmd*^−/−^ mice were fixed and stained with hematoxylin and eosin (H&E). Sections from all infected animals showed areas of consolidated lung tissue indicative of cellular infiltration and inflammation that was absent in noninfected control tissue (Fig. 3*A*). However, WT and *Gsdmd*^−/−^ lung sections showed more severe tissue consolidation and cell infiltration throughout a greater portion of the lung than that seen in *Casp11*^−/−^ mice. We thus quantified cell area versus airway space to determine cellularity scores indicative of pathology for tissue sections from individual mice (26). We observed significantly decreased SARS–CoV-2–induced lung pathology in *Casp11*^−/−^ mice compared to WT and *Gsdmd^−/−^* mice (Fig. 3 *A* and *B*), correlating with the preservation of *Casp11*^−/−^ body weight and their faster recovery (Fig. 2*A*).

Guided by our transcriptomic results indicating that a critical subset of inflammatory mediators are controlled by CASP11 (Fig. 2*F*), we measured levels of CXCL1, IL-1β, and IL-6 by enzyme-linked immunosorbent assay (ELISA) in lung homogenates from infected animals at 2 and 4 d after infection (Fig. 3 *C* and *D*). IL-1β was lower in the lungs of both *Casp11*^−/−^ and *Gsdmd*^−/−^ mice at 2 d after infection when compared to WT (Fig. 3*C*). Moreover, IL-1β staining in lung tissue sections revealed more IL-1β in WT than that observed in *Casp11*^−/−^ mice (*SI Appendix*, Fig. 5). Similarly, the production of CXCL1 was dependent on *Casp11* at both time points, though this was independent of *Gsdmd* (Fig. 3 *C* and *D*). Average levels of IL-6 were partially decreased in *Casp11^−/−^* lungs, with a statistically significant difference between WT and *Casp11^−/−^* lungs at day 4 (Fig. 3 *C* and *D*). These results corroborate and expand our day 2 transcriptomic analysis in which expression of *Il1b* and *Cxcl1* was decreased (Fig. 2*F*) and demonstrate that production of a critical subset of inflammatory mediators in the lung is dependent on CASP11 during SARS–CoV-2 infection.

To determine the role of CASP11 in the response of lung macrophages to SARS–CoV-2, we purified mature primary macrophages from lungs of WT and *Casp11*^−/−^ mice and infected them with SARS–CoV-2 MA10. Culture supernatants and cellular RNA were collected and measured for IL-1β, IL-6, and CXCL1 protein and transcript levels, respectively. Compared with noninfected cells, CXCL1 protein and RNA transcripts were detected at high levels upon infection of WT macrophages but were poorly produced by *Casp11^−/−^* cells (Fig. 3 *E* and *F*). Interestingly, IL-1β transcripts were also induced in a CASP11-dependent manner, but secreted protein was not detected in either group (Fig. 3 *E* and *F*). Distinctly, protein and transcript levels of IL-6 did not significantly differ between WT and *Casp11^−/−^* cells (Fig. 3 *E* and *F*). These results confirm our in vivo measurements and further demonstrate that CASP11 is an important cellular regulator of specific cytokines and chemokines, including CXCL1 and IL-1β, in response to SARS–CoV-2.

### CASP11 Promotes Lung Neutrophil Responses during SARS–CoV-2 Infection.

To better understand the biological processes regulated by *Casp11*, we further analyzed the functional gene enrichment categories of the 236 genes most down-regulated (LFC less than −0.58; *P* value <0.05) in *Casp11^−/−^* lungs. A striking enrichment of neutrophil-related gene signatures emerged that included neutrophil-specific markers (e.g., *Cd177* and *Cxcr2*), neutrophil degranulation genes (e.g., *Pglryp1*, *Ckap4*, *Adam8*, and *Plac8*), and neutrophil complement receptors (*Itgam* and *Itgax*), among others (Fig. 3*G* and *SI Appendix*, Fig. 4*D*). Additionally, genes associated with the response to tissue damage from neutrophils (*Slpi* and *Lair1*) were also decreased in the absence of *Casp11* relative to WT lungs (Fig. 3*G*). These results are consistent with decreased gene expression for the neutrophil chemoattractant CXCL1 (Fig. 3 *C* and *D*), as well as with previous reports of neutrophil regulation by CASP11 through effects on actin (9, 24).

Notably, expression of these neutrophil signature genes in *Gsdmd^−/−^* lungs was less affected than in *Casp11^−/−^* lungs (Fig. 3*G*), though other genes that are down-regulated in the absence of *Gsdmd* were associated with dysregulation of other immune pathways (*SI Appendix*, Fig. 4*E*). Conversely, analysis of genes up-regulated in the absence of *Casp11* revealed a putative association with muscle-specific pathways (*SI Appendix*, Fig. 4*F*), while genes most up-regulated in *Gsdmd^−/−^* lungs were not enriched for any specific functional pathways. Overall, these analyses most prominently demonstrate that CASP11 is required for robust production of specific inflammatory mediators as well as neutrophil recruitment and functions in the lung during SARS–CoV-2 infection.

To further examine the role of neutrophils in SARS–CoV-2 infection, lung sections from WT, *Casp11^−/−^*, and *GsdmD^−/−^* mice were stained for the neutrophil marker Ly6G (Fig. 3*H*). Quantification of Ly6G-positive cells demonstrated fewer neutrophils in *Casp11^−/−^* and *GsdmD^−/−^* lung sections when compared to WT, with a statistically significant difference seen when comparing WT and *Casp11^−/−^*, but not between *Casp11^−/−^* and *GsdmD^−/−^*, mice (Fig. 3*I*). These findings were corroborated by flow cytometric analysis quantifying the percentage of Ly6G^high^ neutrophils among the CD45^+^ immune cells in lung single-cell suspensions from SARS–CoV-2–infected WT, *Casp11^−/−^*, and *GsdmD^−/−^* mice (Fig. 3 *J* and *K*).

One of the main neutrophil-mediated functions is formation of neutrophil extracellular traps (NETs), which contain released chromatin that can immobilize pathogens and trigger immunothrombosis, especially during SARS–CoV-2 infection through platelet–neutrophil interactions (27). We were unable to detect direct infections of neutrophils with SARS–CoV-2 in pilot experiments. Thus, to determine if CASP11 and GSDMD modulate neutrophil functions during SARS–CoV-2 infection, WT, *Casp11^−/−^*, and *Gsdmd^−/−^* neutrophils were treated with phorbol myristate acetate (PMA) (Fig. 4 *A* and *B*) or culture supernatants from WT epithelial cells infected with SARS–CoV-2 to simulate the virus-induced inflammatory milieu (*SI Appendix*, Fig. 6). *Casp11^−/−^* neutrophils were largely defective in forming NETs in response to all conditions compared to WT neutrophils, which formed NETs in response to all conditions (Fig. 4 *A* and *B* and *SI Appendix*, Fig. 6). Together, our data demonstrate that lungs of SARS–CoV-2–infected *Casp11^−/−^* mice contain fewer neutrophils than infected WT lungs, and *Casp11^−/−^* neutrophils largely fail to undergo NETosis.

### The Lack of CASP11 Reduces VWF Levels and Increases Vascular Integrity in Response to SARS–CoV-2.

SARS–CoV-2 infection is accompanied by long-term sequelae mediated in part by vascular damage and thrombosis (28). Given that we noted decreased neutrophil gene signatures in *Casp11^−/−^* lungs upon infection and since tissue infiltration by neutrophils can activate blood clotting cascades and thrombosis (29, 30), we examined whether the production of VWF, a marker for endothelial damage, which is essential to thrombus formation, is regulated by CASP11. Using RNAscope ISH technology, we found significantly more blood vessels expressing VWF messenger RNA (mRNA) in the lung vascular architecture of SARS–CoV-2–infected WT mice when compared to *Casp11^−/−^* lungs at day 4 after infection (Fig. 4*C* and *SI Appendix*, Fig. 7). Immunoblot analysis of lung homogenates confirmed that VWF was significantly lower in lungs of *Casp11*^−/−^ mice when compared to WT lungs (Fig. 4 *D* and *E*). Notably, lung sections from SARS–CoV-2–infected *Gsdmd^−/−^* mice showed more staining for VWF than *Casp11^−/−^* mice (Fig. 4*C*). We thus conclude that CASP11 is required for the accumulation of VWF in the lungs during SARS–CoV-2 infection. Furthermore, we examined the expression of Kruppel-Like Factor 2 (KLF2) in WT and *Casp11^−/−^* SARS–CoV-2–infected lungs since KLF2 is an endothelial cell protective transcription factor that exerts anti-inflammatory and antithrombotic functions and maintains the integrity of the vasculature. We found that KLF2 expression was significantly reduced after SARS–CoV-2 infection in WT lungs but was largely preserved in *Casp11^−/−^* infected lungs (Fig. 4*F*). To confirm the source of VWF, lung sections from *Casp11^−/−^* and *Gsdmd^−/−^* mice were processed for the simultaneous detection of endothelial marker vascular endothelial growth factor (VEGF) receptor 1 (FLT1) and VWF mRNA (31). We found that VWF RNA colocalized with FLT1, which was also up-regulated in WT and *Gsdmd^−/−^* but not *Casp11^−/−^* lung sections (Fig. 4*G* and *SI Appendix*, Fig. 8). Moreover, we examined the vascular architecture in the cleared lungs of SARS–CoV-2–infected mice by using fluorophore-conjugated albumin and tissue clearing (*SI Appendix*, Fig. 9). The vascular tracing revealed distinctive vascular features in WT SARS–CoV-2–infected lungs with pronounced vascular thickening, obliteration, and angiogenesis/neovascularization (Fig. 4*H*). In stark contrast, *Casp11^−/−^* infected lung vasculature did not show these abnormalities, indicating less endothelial damage/dysfunction (Fig. 4*H*). Taken together, these findings indicate that CASP11 contributes to instigation of the coagulation cascade and induction of vascular changes in the lung during SARS–CoV-2 infection.

## Discussion

The medical and research communities have met challenges in identifying specific inflammatory mediators that can be targeted to ameliorate SARS–CoV-2 pathogenesis without impairing beneficial aspects of the immune response, such as viral clearance. A major impediment to mechanistic research in this regard has been the difficulty in infecting mouse models with SARS–CoV-2. Here, we utilized the mouse-adapted SARS–CoV-2 (strain MA10) (20) that was plaque purified, grown in Vero-TMPRSS2 cells, and sequenced to ensure that it lacks the attenuating tissue culture adaptations present in stocks of the virus grown in standard Vero cells, the most commonly used cell line for SARS–CoV-2 propagation (32). Our extensive purification regimen allowed us to achieve measurable pathogenicity in C57BL/6 mice and to infect gene knockout animals for mechanistic research in vivo. Notably, mouse-adapted SARS–CoV-2 strains are emerging as models of choice for studies of viral pathogenicity in mice, though the lack of lethality in this model may not fully phenocopy fatal human disease. The K18-hACE2 mouse model also gained prominence early in the COVID-19 pandemic as an in vivo model susceptible to lethal SARS–CoV-2. However, in this system, hACE2 expression is driven by a keratin promoter, thus likely altering viral tropism, particularly in immune cell subsets where keratin promoter activity may not mirror endogenous ACE2 promoter activity. Thus, the mouse-adapted virus system employed in our studies is particularly advantageous for examining immune-mediated pathogenesis in the lung.

The active inflammasome complex has been implicated in many disease conditions and infections, including SARS–CoV-2 (7, 13). Cell culture experiments identified a minor role for the canonical inflammasome member caspase-1 (CASP1) in SARS–CoV-2 infection (13). CASP11, a member of the noncanonical inflammasome, has not been previously investigated in this context in vitro or in vivo. CASP11 is weakly expressed by resting cells, yet it is induced by bacterial infection and several cytokines (24, 33, 34). We mined available clinical data and found that the expression of human CASP4 and CASP5 in COVID-19 testing swab material correlates with the severity of SARS–CoV-2 infection. Additionally, we found that the expression of CASP4 is elevated in lung sections of SARS–CoV-2 patients. Similarly, mouse CASP11 is up-regulated in the lungs of WT mice in response to SARS–CoV-2. We previously reported that CASP11 restricts *Legionella pneumophila* and *Burkholderia cenocepacia* infections by regulating actin dynamics (9). CASP11 recognizes bacterial LPS in the cytosol, leading to downstream activation of CASP1 and IL-1β (35). However, the role of CASP11 is not restricted to gram-negative bacteria that produce LPS (9), since we found that CASP11 is exploited by the gram-positive bacteria methicillin-resistant *Staphylococcus aureus* to survive in macrophages (16). In these cases, CASP11 regulates the functions of actin machinery to affect vesicular trafficking and cell migration. While it is possible that reduced neutrophil infiltration in SARS–CoV-2–infected *Casp11^−/−^* lungs is due to reduced cytokine and chemokine levels in the lungs, we have also previously shown that even with exogenous addition of chemoattractants, *Casp11^−/−^* immune cells, particularly neutrophils, fail to travel to the inflammation site due to an inherent defect in cell movement (24). Our lung histology and flow cytometry data show that neutrophil reduction in *Casp11^−/−^* and *Gsdmd^−/−^* mice is comparable, yet the pathology in these animals is different. Our transcriptional profiling revealed a defect in cytokine responses, cellular recruitment, and immune activation in the absence of CASP11, demonstrating that *Casp11^−/−^* neutrophils may be nonfunctional when compared to WT and *Gsdmd^−/−^* neutrophils, a notion that is supported by the lack of NETosis in *Casp11^−/−^* neutrophils. On the other hand, GSDMD, which is considered the best characterized effector of CASP11 and CASP4 (12), did not contribute to the lung pathology of SARS–CoV-2–infected mice, explaining why clinical trials using GSDMD inhibitors were not successful (13). Hence, our data suggest that CASP11 mediates many functions that are not executed by GSDMD.

While CASP4/11 can recognize cytosolic LPS from bacteria, it is also activated by oxidized phospholipids that are produced in damaged tissues (36). Overall oxidative stress, including oxidized phospholipids, are reportedly up-regulated in COVID-19 patients (37, 38). Remarkably, oxidized phospholipids induce a CASP4/11-dependent cytokine response without inducing GSDMD-dependent cell death (36), thus mirroring our in vivo results in which *Casp11^−/−^* mice fare better during infection while *Gsdmd^−/−^* mice do not. Thus, it is plausible that CASP4/11 is activated by oxidized phospholipids during infection. It is also possible that CASP4/11 may recognize as yet unidentified products of SARS–CoV-2 replication. Alternatively, high expression of CASP4/11 may result in its auto-activation in the absence of a ligand, as is observed upon its overexpression in vitro. Indeed, CASP4/11 levels can be highly induced by multiple cytokines, including IFNs, present in the SARS–CoV-2–infected lung (36, 39). In sum, whether up-regulated CASP4/11 requires an activation signal and the molecular identity of that signal remains unknown and will be important areas of future research.

The lungs of human patients infected with SARS–CoV-2 show diffuse immune cell infiltration, alveolar damage, alveolar edema and proteinaceous exudates, and destruction of endothelial cells, indicative of acute respiratory distress syndrome (1, 2). Similar findings are detected in WT and *Gsdmd*^−/−^ mice, while lung morphology appears healthier in *Casp11*^−/−^ mice after SARS–CoV-2 infection. In addition, there is less weight loss, with fast recovery to normal weight, in *Casp-11*^−/−^ mice compared with WT and *Gsdmd*^−/−^ mice, which are slower to recover. Importantly, the differences in disease severity are not due to changes in viral burden among different genotypes. This is consistent with a lack of changes in global ISG expression in WT versus *Casp11*^−/−^ or *Gsdmd^−/−^* lungs, which are genes implicated in viral resistance and clearance. Instead, we observed reduced inflammation and lung pathology dependent on CASP11 irrespective of viral loads. In *Casp11^−/−^* but not *Gsdmd^−/−^* SARS–CoV-2–infected mice, chemokines including *Cxcl1*, *Cxcl2,* and *Cxcl14*, which are involved in neutrophil and monocyte recruitment (40), were significantly down-regulated. However, there was no significant difference in expression of IL-1β between *Casp11^−/−^* and *Gsdmd^−/−^* mice. In vitro, IL-1β was barely detectable in the supernatants of macrophages infected with SARS–CoV-2. This can be explained by a recent publication demonstrating that SARS–CoV-2 nucleocapsid inhibits the cleavage of GSDMD in infected cells and hence prevents the release of IL-1β (41). In addition, our data demonstrate that IL-6 is elevated in infected lungs in a CASP11-dependent manner. IL-6 was identified during the COVID-19 pandemic as being a highly up-regulated mediator of disease severity in ill patients. Moreover, high levels of IL-6 can also activate the coagulation system and increase vascular permeability (3).

Postmortem studies have highlighted disseminated microthrombi that, together with increased mortality, morbidity, and long-term sequelae from SARS–CoV-2 infection, are considered hallmarks of severe COVID-19 (42, 43). Currently, the administration of an anticoagulant such as heparin for all hospitalized COVID-19 patients is associated with lower mortality rates and better prognosis (3). Typically, endothelial activation and damage lead to increased VWF production, and this activates the coagulation cascade, along with extensive NETosis elicited by neutrophils, leading to prothrombotic events (28, 44). Importantly, we found here that lungs from *Casp11^−/−^* mice accumulate significantly less VWF in response to SARS–CoV-2 infection, which is largely confined to what appears to be the lining of blood vessels. In contrast, the distribution of VWF in infected WT lungs was intense and diffuse, suggesting the presence of vascular damage.

To further evaluate endothelial damage, we determined the expression of the transcription factor KLF2. Recent reports have linked the vascular injury that is associated with SARS–CoV-2 to reduction in the expression of KLF2 in lung endothelial cells (45). We found that KLF2 levels are largely preserved in *Casp11^−/−^* lungs but are significantly reduced in WT and *Gsdmd^−/−^* lungs. Moreover, the vascular abnormalities we detected on lung vascular tracing indicate severe endothelial damage and endothelialitis in WT SARS–CoV-2–infected lungs. These vascular features resemble the intussusceptive angiogenesis that has been described in SARS–CoV-2–infected human lungs (46, 47). Importantly, the inhibition of angiogenesis through targeting VEGF has been proven beneficial in patients with severe SARS–CoV-2 (48). Notably, we have found less expression of VEGF receptor 1 (FLT1) with less angiogenesis and neovascularization, which is often induced by hypoxia, in the infected *Casp11^−/−^* lungs compared to WT and *Gsdmd^−/−^* lungs. Our data demonstrate a previously unrecognized function for CASP11 in the promotion of coagulation pathways and endothelial dysfunction that lead to thrombotic events. Together with VWF, D-dimer and complement activation status are considered prognostic indicators of adverse outcomes of COVID-19. Remarkably, these factors emerge from the activation of multiple immunothrombosis-related pathways that we found are governed by CASP11 (*SI Appendix*, Fig. 10). Therefore, targeting CASP11 may be beneficial in preventing cytokine storm and many of the thrombotic complications associated with SARS–CoV-2. Additionally, it is possible that exuberant CASP4/11 expression may serve as an earlier biomarker than those currently available for predicting severe disease, cytokine storm, and thrombosis. It is also important to note that unique pathogenic mechanisms may be at play among individuals experiencing severe COVID-19. It will be essential to understand the interplay of CASP4/11 with the heterogenous comorbidities and deficiencies that are associated with COVID-19 morbidity and mortality in the human population.

Our findings collectively suggest that targeting the CASP11 homolog, human CASP4, during COVID-19 will prevent severe pneumonia, inflammation, tissue damage, and thrombosis as well as accompanying repercussions such as low oxygen, lung failure, need for ventilators, and possibly long-term sequelae. These advantageous effects will be achieved without compromising viral clearance. Targeting CASP4 alone may achieve benefits that exceed and replace the administration of a large number of individual anti-inflammatory agents and antithrombotics given to SARS–CoV-2 patients. Further research is needed to develop therapeutics in this regard.

## Materials and Methods

### Biosafety.

All experiments with live SARS–CoV-2 were performed in The Ohio State University (OSU) BSL3 biocontainment facility. All procedures were approved by the OSU BSL3 Operations/Advisory Group, the OSU Institutional Biosafety Officer, and the OSU Institutional Biosafety Committee.

### Viruses and Titers.

Mouse-adapted SARS–CoV-2 variant strain MA10 (20), generated by the laboratory of Ralph Baric (University of North Carolina, Chapel Hill, NC) was provided by BEI Resources (Cat. #NR-55329). SARS–CoV-2 strain USA-WA1/2020 was also provided by BEI Resources (Cat. #NR-52281). Viral stocks from BEI Resources were plaque purified on Vero E6 cells to identify plaques lacking mutations in the polybasic cleavage site of the Spike protein via sequencing. Nonmutated clones were propagated on Vero E6 cells stably expressing TMPRSS2 (provided by Shan-Lu Liu, OSU, Columbus, OH). Virus aliquots were flash frozen in liquid nitrogen and stored at −80 °C. Virus stocks were sequenced to confirm a lack of tissue culture adaptation in the polybasic cleavage site. Virus stocks and tissue homogenates were titered on Vero E6 cells.

### Mice.

C57BL/6 WT mice were obtained from The Jackson Laboratory. *Casp11*^−/−^ mice were generously given by Dr. Junying Yuan at Harvard Medical School, Boston, MA. *Gsdmd*^−/−^ mice were a gift from Thirumala-Devi Kanneganti at St. Jude Children’s Research Hospital, Memphis, TN. K18-hACE2 mice (49) were purchased from The Jackson Laboratory. All infections were performed intranasally on anesthetized mice with viruses diluted in sterile saline. All mice were housed in a pathogen-free facility, and experiments were conducted with approval from the Animal Care and Use Committee at OSU (Columbus, OH), which is accredited by the American Association for Accreditation of Laboratory Animal Care International according to guidelines of the Public Health Service as issued in the Guide for the Care and Use of Laboratory Animals.

### Derivation of Single-Cell Suspension and Primary Lung Macrophages.

Lungs were perfused with cold phosphate-buffered saline (PBS) to remove circulating intravascular white blood cells. Lungs were dissected into single lobes before being dissociated into single-cell suspension using gentleMACS Octo Dissociator and Miltenyi lung dissociation kit (Miltenyi Biotec, 130–095-927). Red blood cells (RBCs) were lysed by incubating cells in 2 mL ACK buffer for 5 min at room temperature (RT). After RBC lysis, cells were washed in DPBS containing 1% bovine serum albumin (BSA). The single-cell suspension was centrifuged, and the cell pellets were washed twice with PBS. Cell pellets were further suspended in 0.5 mL PBS 1% BSA. This was followed by CD11b magnetic bead (Miltenyi Biotec, 130–049-601) isolation technique to positively select for macrophages expressing the pan-macrophage/monocyte CD11b marker.

### Flow Cytometry.

Single-cell suspension from the previous step was stained with fluorophore-conjugated antibodies for fluorometric analysis as described before (50).

### Murine Tracheobronchial Epithelial Three-Dimensional (3D) Cultures.

Murine trachea and bronchioles were dissected from two mice each of C57BL/6 WT, *Casp11^−/−^*, and *Gsdmd^−/−^*. Isolation of tracheobronchial epithelial cells was as follows. Tissues were washed, and tracheas were incubated overnight in Ham’s F-12, 1% penicillin/streptomycin, 1% amphotericin B (Thermo Fisher Scientific, #15290018) and Pronase from *Streptomyces griseus* (Sigma-Aldrich, #10165921001) solution. Digestion of trachea and bronchioles were neutralized with 10% fetal bovine solution (FBS; Life Technologies, #10438026), and tracheal airway cells were gently scraped. Cells were washed three times in Ham’s F-12, 10% FBS, and 1% penicillin/streptomycin solution and further digested in deoxyribonuclease I solution (Sigma-Aldrich, #DN25-10) in Ham’s F-12 with 10 mg/mL BSA (Thermo Fisher Scientific, #BP9706). Airway cells were then washed with Murine Tracheobronchial Epithelial Cell (MTEC) base medium [1:1 Ham’s F-12: Dulbecco’s modified Eagle’s medium (Thermo Fisher Scientific, #11995065), plus 10% FBS, 1% penicillin/streptomycin, 50 µg/mL gentamicin (Life Technologies, #15710064), and 0.03% wt/vol NaHCO_3_]. Cells were plated in a T25 flask (Thermo Fisher Scientific, #1012610) overnight in MTEC medium at 37 °C, 5% CO_2_. The next day, medium was switched to 1:1 of MTEC and PneumaCult-Ex PLUS medium (StemCell Technologies, #05040) and fed every other day until expansion of cells to ∼80% confluent. Epithelial cells were then trypsinized twice with TrypLE Express (Thermo Fisher Scientific, #12605010) to remove residual fibroblast cells and seeded at a density of 50,000 cells per transwell in Corning 6.5-mm, 24-well transwells (Thermo Fisher Scientific, #07200154) in 1:1 MTEC:PneumaCult-Ex PLUS medium. Cells were fed for 4 to 5 d until airlifted and continued to be grown at air-liquid interface (ALI) with PneumaCult ALI medium (StemCell Technologies, #05001) until fully differentiated (4 wk).

### Immunoblotting.

Protein extraction from lung tissue was performed using TRIzol reagent (Thermo Fisher Scientific, #15596026) according to the manufacturer’s instructions. Equal amounts of protein were separated by sodium dodecyl sulfate–polyacrylamide gel electrophoresis and transferred to a polyvinylidene fluoride membrane. Membranes were incubated overnight with antibodies against CASP11 (Cell Signaling Technology, 14340), VWF (Protein Tech, 11778–1-AP), and β-Actin (Cell Signaling Technology, 3700). Corresponding secondary antibodies conjugated with horseradish peroxidase in combination with enhanced chemiluminescence reagent (Amersham, RPN2209) were used to visualize protein bands. Densitometry analyses were performed by normalizing target protein bands to their respective loading control (β-Actin) using ImageJ software as previously described (24, 51).

### ELISAs.

Cytokine/chemokine ELISAs were performed on lung homogenates or macrophage supernatants using R&D Systems Duoset ELISA kits (IL-6, DY406; IL-1b, DY401; CXCL1, DY453) according to the manufacturer’s instructions.

### Histology.

Lungs were removed from infected mice and fixed in 10% formalin at RT. Sample preparation, processing, H&E staining, and semiquantitative slide evaluation using ordinal grading scales were performed as previously described (51). Lungs used for immunofluorescence (IF) staining and RNAscope ISH technique were embedded in optimal cutting temperature compound (OCT) and flash frozen, while lung tissue used for immunohistochemistry (IHC) was embedded in paraffin blocks.

### IHC and IF Staining for Mouse Tissues.

IF staining of mouse lung sections was performed as previously described (51). Slides were washed three times for 15 min with PBS to remove residual OCT. The sections were then incubated in the blocking solution (PBS containing 10% donkey serum; MilliporeSigma, Cat. #S30-100 mL), 2% BSA (Thermo Fisher Scientific, BP1600-100), and 0.3% Triton X-100 (Thermo Fisher Scientific, BP151-100) for 2 h at RT. Sections were then transferred to blocking solution containing the primary antibody against IL-1β (GeneTex, GTX74034) and incubated overnight at 4 °C. After that, sections were washed with PBS 3X for 15 min each. Then, they were incubated with the blocking solution containing the secondary antibody for 2 h at RT. DAPI (Thermo Fisher Scientific, Cat. #D1306) was added to the staining solution in the last 15 min of incubation at a final concentration (5 μg/mL). Finally, sections were washed with PBS 3X for 15 min. Antifade mounting media (Thermo Fisher Scientific, Cat. #P36934) was added before coverslipping. For IHC, Ly6G (Abcam, ab25377) and SARS–CoV-2 nucleocapsid protein (GeneTex, GTX635686) primary antibodies were used. All the stainings were performed at Histowiz, Inc., Brooklyn, NY, using the Leica Bond RX automated stainer (Leica Microsystems). The slides were dewaxed using xylene and alcohol-based dewaxing solutions. Epitope retrieval was performed by heat-induced epitope retrieval of the formalin-fixed, paraffin-embedded tissue using citrate-based pH 6 solution for 40 min at 95 °C. The tissues were first incubated with peroxide block buffer (Leica Microsystems), followed by incubation with the rabbit Caspase 4 antibody (Novus Bio, NBP1-87681) at 1:700 dilution for 30 min, followed by diaminobenzidine (DAB) rabbit secondary reagents: polymer, DAB refine, and hematoxylin (Leica Microsystems). The slides were dried, coverslipped, and visualized using a Leica Aperio AT2 slide scanner (Leica Microsystems).

### RNAscope ISH.

Lung tissue was fixed and embedded in OCT as described above. Sections of 15-μm thickness were mounted on Plus charged slides. ISH was performed using RNAscope Multiplex Fluorescent Reagent Kit v2 (Advanced Cell Diagnostics, Cat. #323100) as described before (52). All incubations between 40 and 60 °C were conducted using an ACD HybEZ II Hybridization System with an EZ-Batch Slide System (Advanced Cell Diagnostics, Cat. #321710). Slides were washed in PBS twice to remove any residual OCT, then baked at 60 °C for 30 min. Baked slides were subsequently postfixed in cold 10% formalin for 15 min, then washed and treated with hydrogen peroxide solution (10 min at RT; Advanced Cell Diagnostics, Cat. #322335). After being rinsed twice with double-distilled H_2_O, sections were incubated in RNAscope Target Retrieval Solution (98 °C for 5 min; Advanced Cell Diagnostics, Cat. #322001) and rinsed three times. Next, a hydrophobic barrier was created around the tissue using an ImmEdge Pen (Advanced Cell Diagnostics; Cat. #310018), and slides were incubated with RNAscope Protease III (30 min at 40 °C; Advanced Cell Diagnostics, Cat. #322337) and subsequently incubated with RNAscope target probes VWF (Cat. #499111), FLT1 (Cat. #415541-C2), and Casp4/Casp11 (Cat. #589511) for 2 h at 40 °C. Next, slides were washed twice with 1X Wash Buffer (Advanced Cell Diagnostics, Cat. #310091; 2 min/rinse at RT), followed by sequential tissue application of the following: RNAscope Multiplex FL v2 Amp 1 (Advanced Cell Diagnostics, Cat. #323101), RNAscope Multiplex FL v2 Amp 2 (Advanced Cell Diagnostics, Cat. #323102), and RNAscope Multiplex FL v2 Amp 3 (Advanced Cell Diagnostics, Cat. #323103). This was followed by application of RNAscope Multiplex FL v2 HRP C1or C2 (15 min at 40° C; Advanced Cell Diagnostics, Cat. #323104). Finally, Opal dyes (Thermo Fisher Scientific, NC1601877 and NC601878) were then applied, after being (Thermo Fisher Scientific, NC1601877) diluted in RNAscope TSA buffer, (Advanced Cell Diagnostics, Cat. #322809) for 30 min at 40 °C. HRP blocker was subsequently added to halt the reaction. Finally, slides were incubated with DAPI, coverslipped with ProLong Gold Antifade Mountant (Thermo Fisher Scientific, P36930), and stored at 4 °C until image acquisition.

### Confocal Imaging and Analysis.

Fluorescent images were captured on an Olympus FV 3000 inverted microscope with a motorized stage. A 2x objective was used to create a map of the lung section in the X,Y dimension. This was followed by using 20x objective to create a stitched z-stacked 3D panoramic view of the lung section. Images were taken by using the 488-nm, 543-nm, and 405-nm (for DAPI) lasers. Image reconstructions of z-stacks and intensity projection images were generated in IMARIS software (Bitplane, Inc.). *Flt1* mRNA expression was quantified using spot function in IMARIS. Number of cells was also quantified via the spot functions.

### Vasculature Labeling with Conjugated Albumin.

The mouse vasculature was labeled as reported previously (53). Briefly, mice were transcardially perfused with 10% formalin in PBS. Mice were then perfused with 5 mL 0.05% albumin–tetramethylrhodamine isothiocyanate bovine (Sigma-Aldrich, #A2289) in 2% gelatin from porcine skin (Sigma-Aldrich, #G1890). At the time of injection, the temperature of the gel solution was kept at 45 °C. After the heart was clamped, mice were placed on ice to lower the body temperature and allow gel formation. Lungs were postfixed in 10% formalin for 10 d. The unsectioned lungs were then cleared using the advanced CUBIC (Clear, Unobstructed Brain Imaging Cocktails and Computational Analysis) protocol (54) and imaged using a confocal microscope (C2, Nikon).

### NET Formation Assay.

Bone marrow was collected from WT, *Gsdmd^−/−^*, or *Casp11^−/−^* mice, then neutrophils were negatively selected by using the EasySep mouse neutrophil enrichment kit (STEM Cell Technologies, #19762A) and 200,000 neutrophils/well were plated in a 24-well plate on fibronectin-coated glass coverslip. Polymorphonuclear neutrophils were stimulated for 4 h with 100 nM PMA (Sigma-Aldrich, #P8139-10MG) or conditioned media from SARS–CoV-2–infected epithelial cells. The cells were fixed with 4% paraformaldehyde, permeabilized with 0.2% Triton X-100 for 10 min, and blocked with 10% goat serum for 30 min at RT. For the visualization of NETs, neutrophils were stained with rabbit anti-mouse Histone 2b (Abcam, #ab1790), mouse anti–double-stranded DNA (dsDNA) (Abcam, #ab27156), goat anti-rabbit immunoglobulin (Ig)G Alexa Fluor 555 (Thermo Fisher Scientific, #A32732), goat anti-mouse IgG Alexa Fluor 488 (Abcam, #ab150113), and wheat germ agglutinin Alexa Fluor 350 (Thermo Fisher Scientific, #W11263). The coverslips were mounted with Fluoroshield Mounting Medium (Abcam, #ab104135). The cells were visualized by confocal microscopy (Zeiss 800 Confocal microscope). The % of cells producing NETs was calculated per field of view by using Fiji software (NIH). The cells producing NETs were counted when DNA was decondensed and ejected from the cells: *% NETs = (neutrophils with DNA projections × 100)/total number of neutrophils.*

### RNA Sequencing and Data Analysis.

Total RNA was extracted from day 2 SARS–CoV-2 WT, *Casp11*^−/−^, and *Gsdmd*^−/−^ infected lungs by TRIzol reagent (Thermo Fisher Scientific, #15596026) according to the manufacturer’s instructions. RNA cleaning and concentration were done using Zymo Research, RNA Clean & Concentrator-5 kit (Cat. #R1015) following the manufacturer’s protocol. Fluorometric quantification of RNA and RNA integrity analysis were carried out using RNA Biochip and Qubit RNA Fluorescence Dye (Invitrogen). Complementary DNA (cDNA) libraries were generated using NEBNext Ultra II Directional (stranded) RNA Library Prep Kit for Illumina (NEB, #E7760L). Ribosomal RNA was removed using NEBNext rRNA Depletion Kit (human, mouse, rat) (E #E6310X). Libraries were indexed using NEBNext Multiplex Oligos for Illumina Unique Dual Index Primer Pairs (NEB, #644OS/L). Library prep–generated cDNA was quantified and analyzed using Agilent DNA chip and Qubit DNA dye. Ribo-depleted total transcriptome libraries were sequenced on an Illumina NovaSeq SP flow cell (paired-end, 150-bp format; 35 to 40 million clusters, equivalent to 70 to 80 million reads). Library preparation, quality control, and sequencing were carried out at Nationwide Children’s Hospital genomic core.

Sequencing data processing and analysis were performed by the Bioinformatics Shared Resource Group at OSU using previously published pipelines (55). Briefly, raw RNA sequencing data (fastq) were aligned to mouse reference genome (GRCh38) using hisat2 (v2.1.0) (56) and converted to counts using the “subread” package (v1.5.1) (57) in R. In the case of multimapped reads, the primary alignment was counted. Low expressed counts were excluded if more than half of the samples did not meet the inclusion criteria (2 counts per million [CPM]). Data were normalized using “voom,” and statistical analysis for differential expression was performed with “limma” (58). For data visualization, DESeq2 rlog transformation was used for principal component analysis (PCA). Volcano plots were generated with “EnhancedVolcano” and heatmaps were generated with ComplexHeatmap” using R. Functional enrichment was performed with Ingenuity Pathway Analysis (Qiagen) to enrich for IPA Canonical Pathways, “clusterProfiler” to generate enrichment maps (55), and EnrichR (59).

### Single Cell RNA Sequencing Data Mining.

Human lung single-cell expression analysis for *CASP4* and *CASP5* was performed by the Human Protein Atlas version 21.0 (http://www.proteinatlas.org) (18). Mouse lung single-cell expression analysis for *Casp11* was performed on published data (GEO dataset accession No. GSE186360) exactly as previously described (21).

### Statistical Analysis.

Data were analyzed using GraphPad Prism 8.3.0. All figures display mean and SD (SD) or SEM (SEM) from independent experiments, as indicated in the figure legends. Comparisons between groups were conducted with either upaired *t* test or ANOVA followed by Tukey’s multiple comparisons test. Adjusted *P* < 0.05 was considered statistically significant.

## Supplementary Material

Supplementary File

## Data Availability

RNA sequencing data comparing WT, *Casp11^−/−^*, and *Gsdmd^−/−^* lungs are shared through GEO with Accession No. GSE184678.
