# Supplementary material for: Caspase-4/11 exacerbates disease severity in SARS–CoV-2 infection by promoting inflammation and immunothrombosis
Source: Proc Natl Acad Sci U S A. 2022 May 19;119(21):e2202012119. doi: 10.1073/pnas.2202012119 (PMC9173818; doi:10.1073/pnas.2202012119)
Supplement: Supplementary File [file pnas.2202012119.sapp.pdf]

Supplementary figures

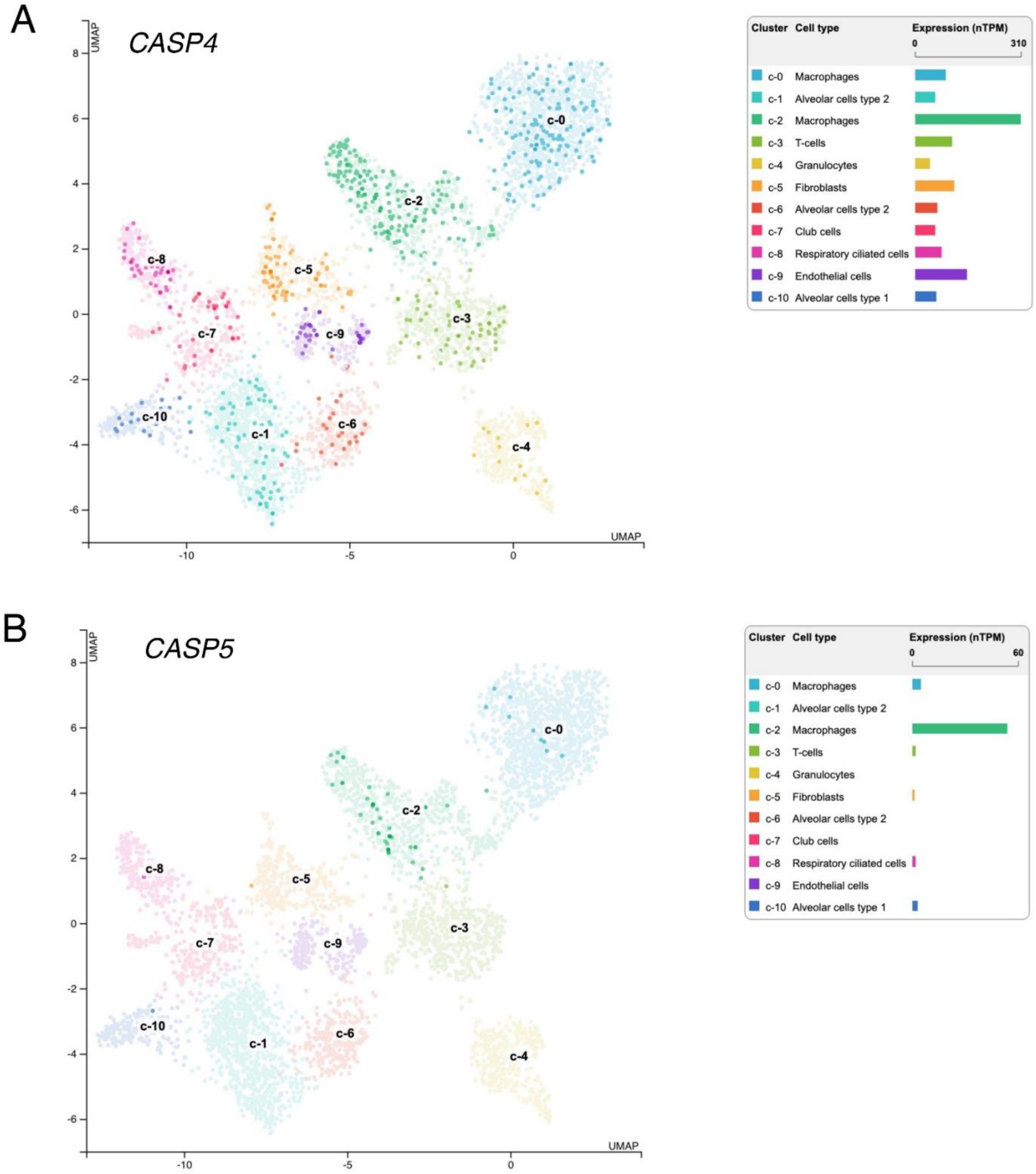

Supplementary Fig. 1: Cell type-specific expression profiles of CASP4 and CASP5 genes in the human lung. Single cell expression data contained in the Human Protein Atlas version 21.0 was mined to identify cell types in the human lung that express **A**, CASP4 (https://www.proteinatlas.org/ENSG00000196954-CASP4/single+cell+type/lung) and **B**, CASP5 (https://www.proteinatlas.org/ENSG00000137757-CASP5/single+cell+type/lung).

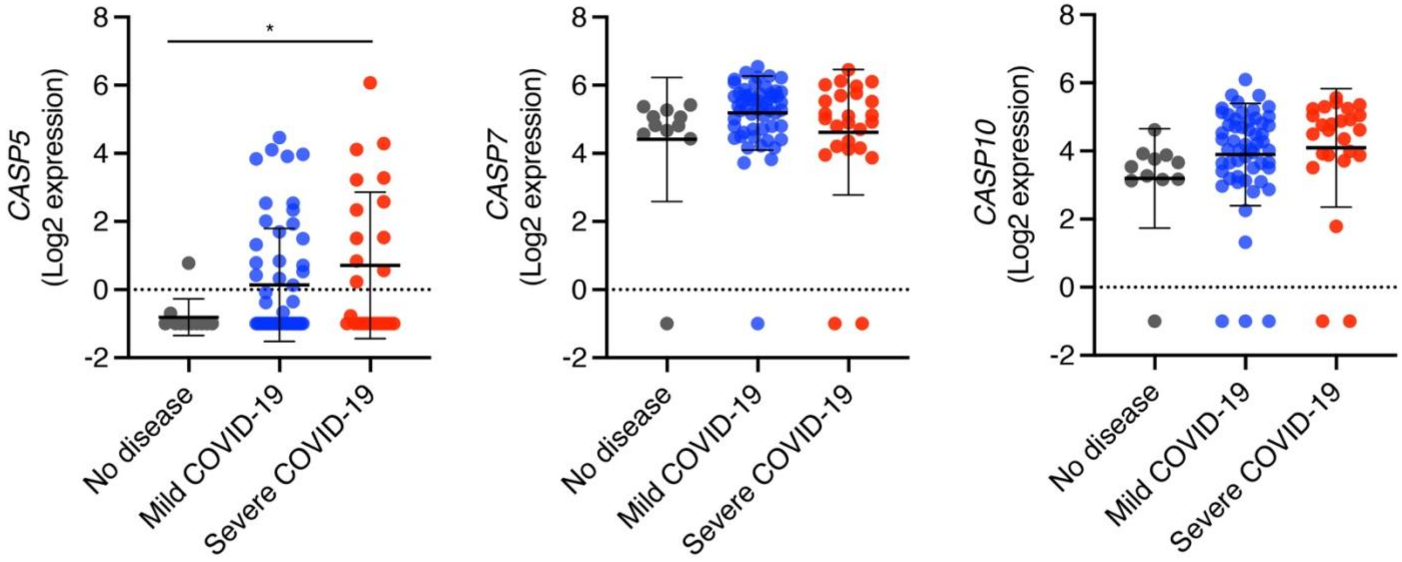

Supplementary Fig. 2: Human caspase expression levels in nasopharyngeal swab samples. CASP5, CASP7, and CASP10 expression levels from RNA sequencing of nasopharyngeal swab samples from patients with no disease, mild SARS-CoV-2, or severe SARS-CoV-2 [GSE145926], one way ANOVA with Tukey's multiple comparisons test, *P<0.05.

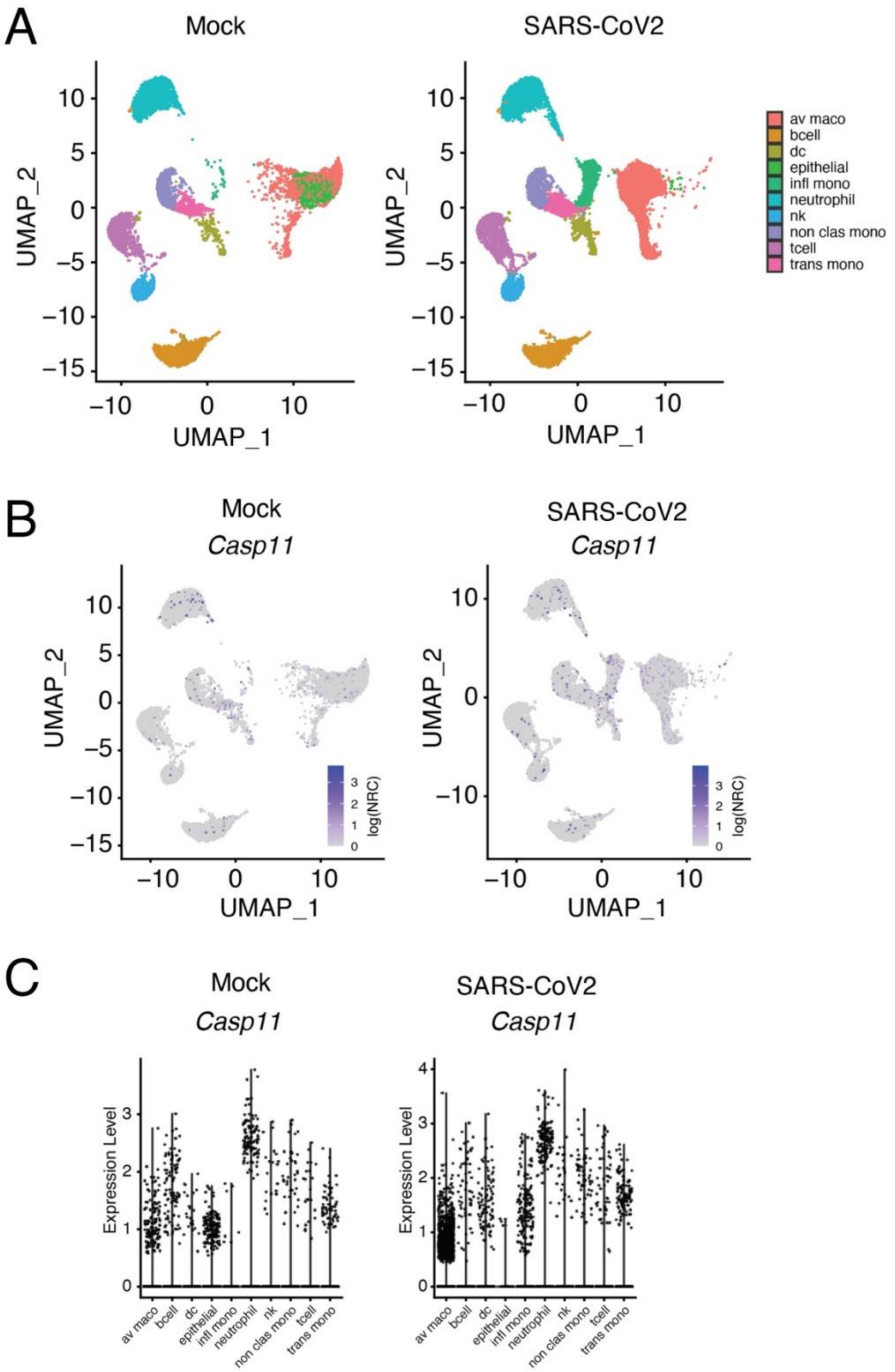

Supplementary Fig. 3: Expression profiles of Casp11 in murine lung immune cells. Analysis of single cell gene expression from mock and SARS-CoV-2 infected lungs. **A**, UMAP analysis of cell subsets in mock lungs (left) and lungs infected with SARS-CoV2 (right). **B**, Log normalized read counts (NRC) of Casp11 in single cells derived from lungs of mock (left) and SARS-CoV-2 infected (right) mice. **C**, Violin plots of the relative expression of Casp11 across cell types identified in mock (left) and SARS-CoV-2 infected lungs (right).

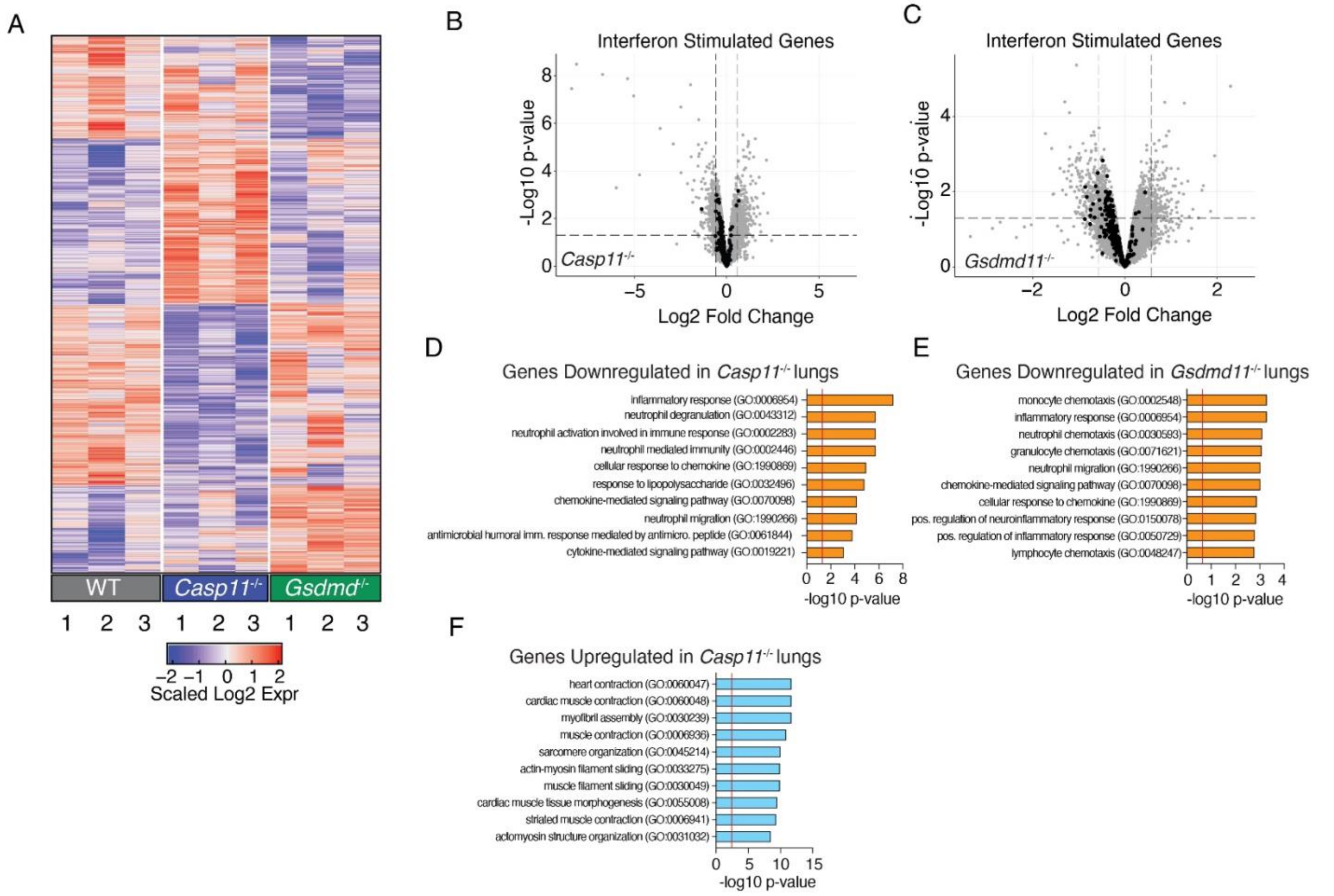

Supplementary Fig. 4: Changes in inflammatory responses in *Casp11*$^{-/-}$ and *Gsdmd*$^{-/-}$ SARS-CoV-2-infected lungs. A, Heat map of significant gene expression changes (p-value <0.05). Depicted genes were chosen based on comparisons relative to WT. Color indicates relative upregulation (red) or downregulation (blue) in gene expression. **B-C,** Statistical analysis of ISG expression in *Casp11*$^{-/-}$ and *Gsdmd*$^{-/-}$ infected lungs relative to WT. Each point represents transcripts within the dataset. 300 IFNb-responsive ISGs are highlighted in black. Dashed lines represent LFC and p-value cutoffs (LFC |0.58| and p-value 0.05). **D,** Functional enrichment analysis of the top 236 downregulated genes in *Casp11*$^{-/-}$ SARS-CoV-2-infected lungs relative to infected WT. Red vertical line represents threshold of significance p-value 0.05. **E,** Functional enrichment analysis of the top 224 downregulated genes in *Gsdmd*$^{-/-}$ infected lungs relative to WT infection. Red vertical line represents threshold of significance (p-value <0.05). **F,** Functional enrichment analysis of 328 upregulated genes *in Casp11*$^{-/-}$ infected lungs relative to WT infection.

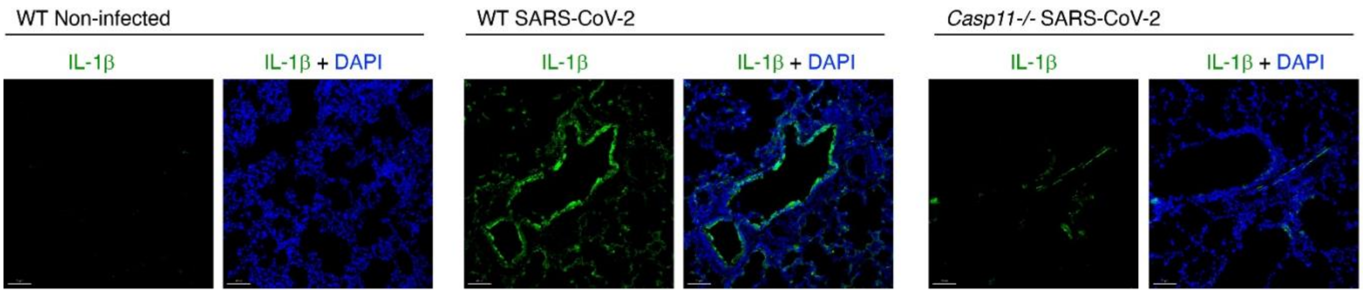

Supplementary Fig. 5: IL-1b production during SARS-CoV-2 infection is decreased in the absence of *Casp11*. WT and *Casp11*$^{-/-}$ mice were infected with SARS-CoV-2 (MA10, 10^5 pfu). Lungs were collected at day 4 post-infection. Lung tissue was sectioned and stained for IL-1β (green), and DAPI (blue).

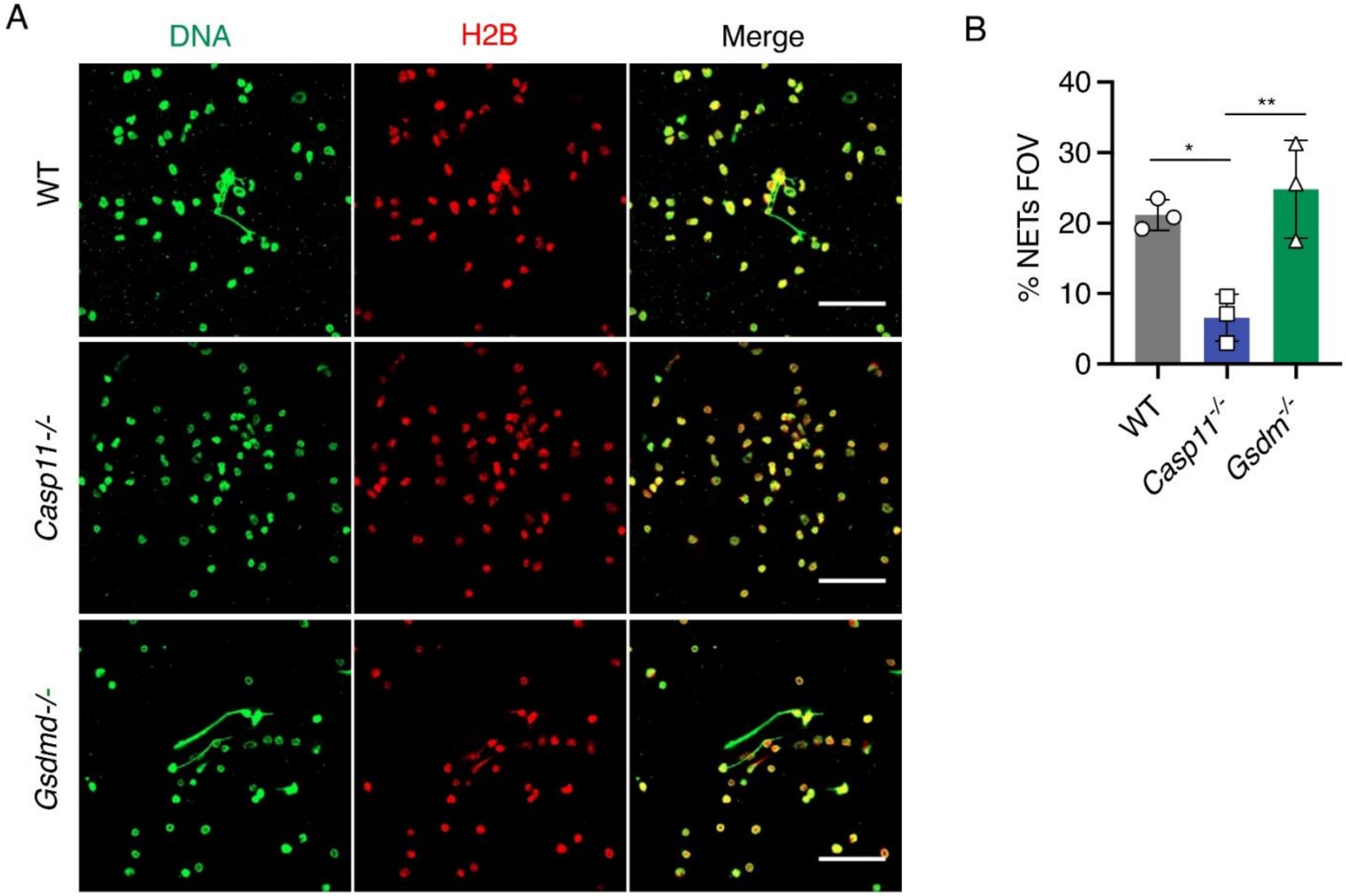

Supplementary Fig. 6: *Casp11*$^{-/-}$ neutrophils are impaired in NET formation. A, Neutrophils from WT, *Casp11*$^{-/-}$ and *Gsdmd*$^{-/-}$ mice were treated with supernatants of WT epithelial cells infected with SARS-CoV-2 MA10 (MOI 1) and NET formation was visualized by staining with anti-mouse Histone 2b (red) and anti-dsDNA (green). Images were captured at 60x magnification. **B,** Percent of cells undergoing NETosis from images as in **A** averaged from 10 fields of view (FOV) for each experimental replicate, one way ANOVA with Tukey's multiple comparisons test, *P<0.05, **P<0.005.

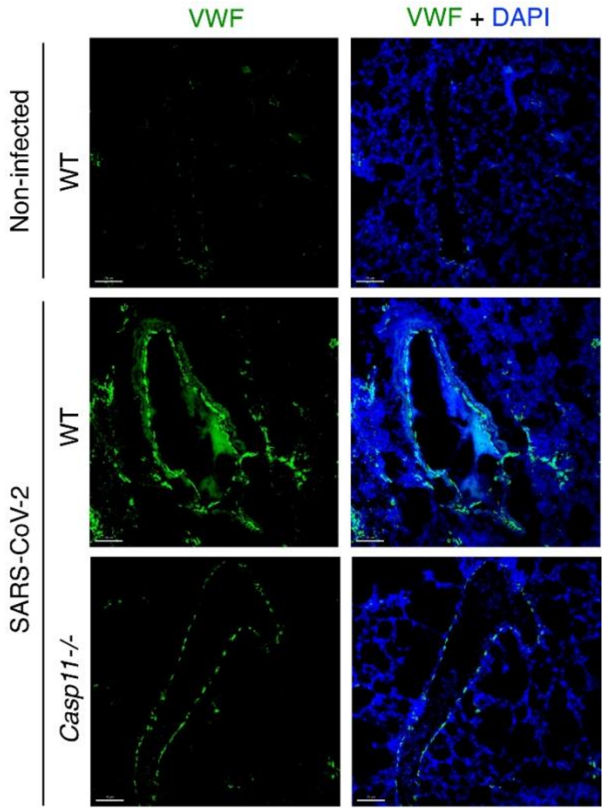

Supplementary Fig. 7: VWF accumulation at blood vessels during SARS-CoV-2 infection is decreased in the absence of *Casp11*. Mice were infected with SARS-CoV-2 (MA10, 10^5 pfu). Lungs were collected at day 4 post-infection. RNA of *VWF* was detected by RNAscope *in situ* hybridization (green) and nuclei were staining with DAPI (blue). Images were captured by a 20x objective. Full lung stitched images are shown in main text **Fig. 4B**.

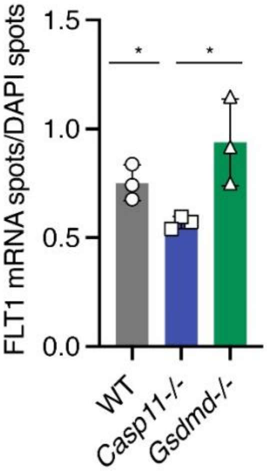

Supplementary Fig. 8 FLT1 is downregulated in *Casp11*$^{-/-}$ SARS-CoV-2-infected lungs.
Quantification of *in situ* hybridization RNAscope staining of endothelial VEGF receptor subtype 1 (FLT1) in lung sections. Mice were infected with SARS-CoV-2 (MA10, 10^5 pfu). Lungs were collected at day 4 post-infection. Original Images were captured by a 20x objective in a 3D stitched panoramic view representing the whole lung in x,y and z in lung sections of samples described in **4f** . DAPI and FLT1 mRNA spots were quantified by using the spot function in IMARIS software. Unpaired t test. *p<0.05

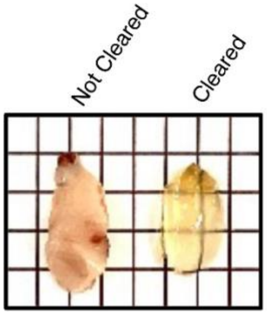

Supplementary Fig. 9: Example clearing of lungs for vascular imaging. Representative photograph of lungs with and without tissue clearing.

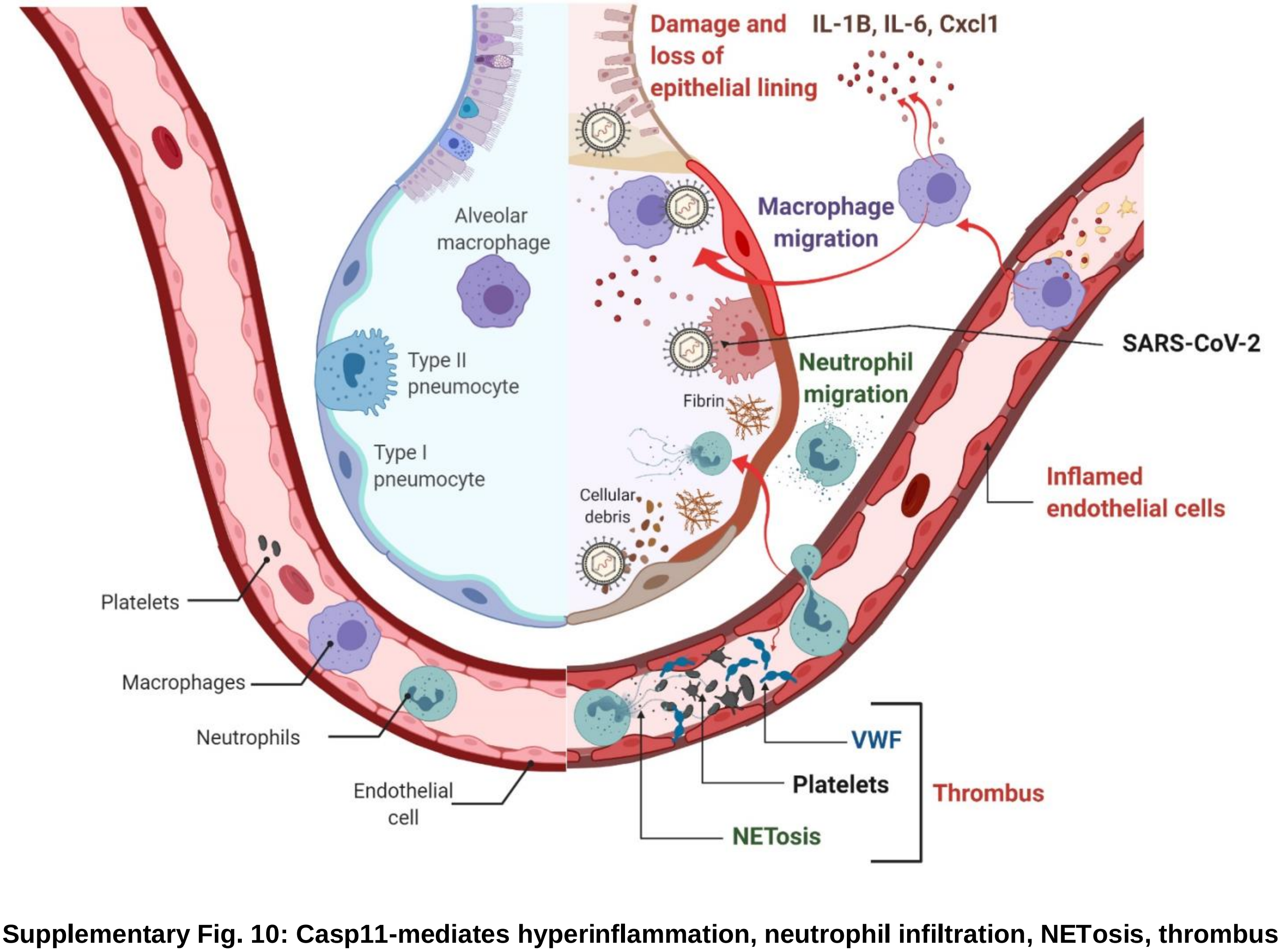

Supplementary Fig. 10: Casp11-mediates hyperinflammation, neutrophil infiltration, NETosis, thrombus formation and vascular damage during SARS-CoV-2 infection.
